# The Effect of Response Conditions on Food Images-Evoked Emotions Measured Using the Valence × Arousal Circumplex-Inspired Emotion Questionnaire (CEQ)

**DOI:** 10.3390/foods12112250

**Published:** 2023-06-02

**Authors:** Han-Seok Seo, Lydia Rockers, Young-Gab Kim

**Affiliations:** 1 Department of Computer and Information Security, and Industry Academy Cooperation Foundation, Sejong University, 209 Neungdong-ro, Gwangjin-gu, Seoul 05006, Republic of Korea; 2 Department of Food Science, University of Arkansas, 2650 North Young Avenue, Fayetteville, AR 72704, USA; lrockers@uark.edu; 3 Department of Computer and Information Security, and Convergence Engineering for Intelligent Drone, Sejong University, 209 Neungdong-ro, Gwangjin-gu, Seoul 05006, Republic of Korea

**Keywords:** emotion, food, image, check-all-that-apply, valence, arousal, CEQ

## Abstract

In 2020, a single-response-based, valence × arousal circumplex-inspired emotion questionnaire (CEQ) was developed. Using a between-participants design, previous studies have found that a multiple response (MR) condition better discriminated test samples (e.g., written food names) based on their evoked emotions than a single response (SR) condition. This research, comprising Studies 1 and 2, aimed to determine the effect of response conditions (i.e., SR *vs*. MR) on emotional responses to food image samples, using a within-participants design. In Study 1, 105 Korean participants were asked to select a pair of emotion terms (i.e., SR condition) or select all pairs representing their evoked emotions (i.e., MR condition) from a list of 12 pairs of emotion terms of the CEQ, in response to the 14 food images. Both SR and MR conditions were tested within a remote (online) session. To minimize both a potential carry-over effect of the “within-participants design” and an influence of environmental factors in the remote testing, Study 2 asked 64 U.S. participants to do so over two separated sessions on two different days in a controlled laboratory setting. In both Studies 1 and 2, participants selected the CEQ’s emotion-term pairs in the MR condition more frequently than in the SR condition, leading to the MR condition’s higher capacity to discriminate test samples. While the configurations of the correspondence analysis biplots drawn in the SR and MR conditions were similar, those in the MR condition were more likely to be similar to the configurations of the principal component analysis biplots drawn from the ratings of valence and arousal for food image samples. In conclusion, this study provides robust empirical evidence that the MR condition can perform better in capturing sample differences in food-evoked emotions, while the SR condition is also effective in characterizing emotional profiles of test samples. Our findings will provide practical insights to sensory professionals, enabling them to effectively leverage the CEQ or its variants when measuring food-evoked emotions.

## 1. Introduction

The intensity of sensory attributes or consumer acceptance alone is not a reliable predictor of market success for new food products [1]. Previous studies have highlighted the limitations of relying solely on the results of typical sensory tests (e.g., consumer acceptance or preference tests) to accurately predict purchase-related behaviors [2,3,4,5,6,7]. Although there are numerous non-sensory factors related to food choice [5], food-evoked emotions, characterized as “brief but intense physiological and/or mental reactions to a food or beverage item” [2,8,9], have also been identified as crucial factors associated with consumer acceptance and behavior in food-related contexts [3,4,7,8,9,10,11,12,13]. Previous studies have notably shown that profiles of food-evoked emotions or specific emotional attributes differentiated test samples more effectively than hedonic ratings [11,14,15]. On the other hand, while some emotion-related terms have been linked to acceptability consistently, other emotion-related terms have not been associated with acceptability of food samples [11,15,16,17]. These observations suggest that food-evoked emotions may provide “added values” beyond simply liking when predicting consumers’ food choice [3,4]. In fact, combining measurements of both sensory and emotional responses to food samples has been found to produce higher predictability of consumers’ choice or purchase-related behavior than using either one by itself [7,18,19]. Because researchers have identified the impact and applicability of food-evoked emotions in the food industry, there has been a dramatically growing interest in this area over the past five years. According to a recent systematic review of 193 peer-reviewed articles on this topic published between January 1997 and March 2021, more than 60% of them were published between January 2017 and March 2021 (*n* = 117), representing a significant increase by 13 times compared to the number of articles published between 2004 and 2008 (*n* = 9) [13]. Over the past decade, numerous review articles on food-evoked emotions have also highlighted the growing attention and popularity of the topic in the field of sensory and consumer sciences [8,12,13,20,21,22,23,24,25,26,27,28,29,30].

Apart from active debates on the definitions and dimensions of food-evoked emotions [12,13,28], there is another main question related to how we measure food-evoked emotions. From the 193 peer-reviewed articles published between January 1997 and March 2021, Low et al. [13] identified 55 different methods used for measuring food-evoked emotions as functions of level of measurement (i.e., cognitive, behavioral, and physiological) and level of emotional processing (i.e., explicit and implicit). Almost half (*n* = 26, 47.3%) of the 55 different methods were classified as cognitively explicit instruments (e.g., using emotion lexicon), and more than 70% (*n* = 140, 72.5%) of the 193 studies employed such a cognitively explicit instrument for measuring food-evoked emotions [13], (see also [28]). As such, the cognitively explicit instrument, mainly using self-reported emotion questionnaires, has become the most popular method for assessing food-evoked emotions. There is also general agreement that using a self-reported emotion questionnaire, i.e., asking participants to identify terms representing their emotional responses to test samples and/or rate intensities of the emotional attributes on scales, is the most effective method for measuring food-evoked emotions [13,26,29,30,31]. Specifically, cognitively explicit measurement in the form of a self-reported questionnaire offers a comprehensive approach to measuring food-evoked emotions [26]. This method also provides simplicity and ease of implementation and analysis, making it a cost-effective and time-efficient option [26,29,30,31]. Although various types of self-reported questionnaires have been developed for measuring food-evoked emotions [29,30,31,32], the EsSense Profile^®^ [2], comprising 39 emotion terms, has emerged as the most commonly used questionnaire [13].

**Figure 1:**
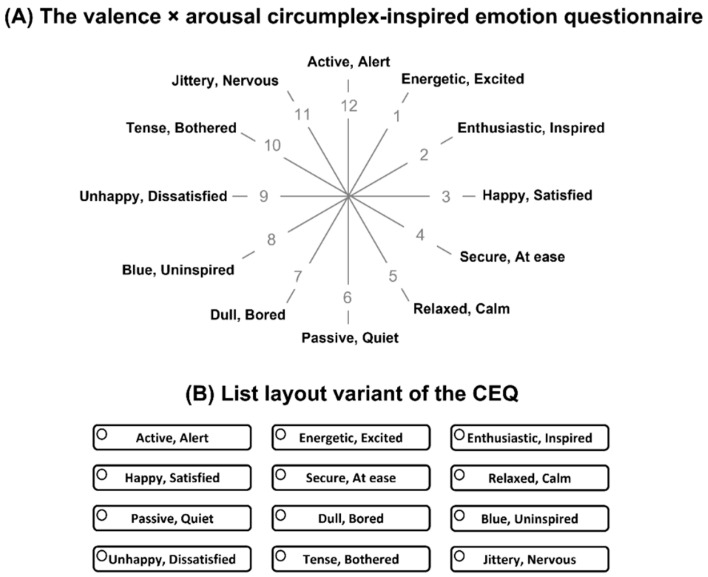
The circular layout (**A**) of the valence × arousal circumplex-inspired emotion questionnaire (CEQ) [35] and its list layout variant (**B**) used in the present study. Note: the numerical values (1 to 12) shown in the circular layout (**A**) are not used in the original form of the CEQ but are shown for reference [38]. Figure 1A was adapted from Figure 1 of Jaeger et al. [38], with permission from the corresponding publisher. In the present study, the order of the 12 pairs of emotion terms in the list layout variant was randomized across food image samples and participants.

While the self-reported emotion questionnaire is a recommended tool for assessing food-evoked emotions [26,29,30,31], its formats have evolved over time to better conceptualize and time-efficiently capture emotional responses to foods. For example, several modified versions of existing emotion-related questionnaires, such as the EsSense Profile^®^, have been introduced [33,34], including the EsSense25 comprised of 25 emotion terms [33]. New types of self-reported emotion questionnaires have also been developed in recent years [35,36,37]. In 2020, Jaeger et al. [35] developed a single-response questionnaire inspired by a circumplex model of core affect, referred to as “the valence × arousal circumplex-inspired emotion questionnaire” (CEQ). The wheel-shaped questionnaire consists of 12 pairs of emotion terms (e.g., enthusiastic, inspired) that represent two dimensions, “valence” and “arousal”. As shown in Figure 1, it employs 12 axes that represent combinations of the two dimensions. Unlike the EsSense Profile^®^ questionnaire, the CEQ has balanced numbers of pleasant (positive) and unpleasant (negative) emotion-related terms and includes both dimensions of valence and arousal. Another distinctive feature of the CEQ is its inclusion of 12 domains related to valence and arousal, and participants are asked to select which domain best represents how they feel and to circle the corresponding pair of emotion terms [35]. In their study using the CEQ, Jaeger et al. [38] tested whether emotional responses to 15 food names might differ as a function of question layout (i.e., circular *vs*. conventional list) and response format (i.e., single response *vs*. multiple response). The list layout of the emotion terms did not differ from the circular layout with respect to the frequency of citation for emotion terms, the percentage of significant pairwise sample comparisons, and the configurations of the two-dimensional biplots drawn by correspondence analyses. However, when multiple responses were allowed by using a Check-All-That-Apply (CATA) procedure, participants selected more than a single pair of emotion terms, thereby increasing the number of significant pairwise sample comparisons compared to when only a single response was allowed. In their follow-up studies, Jaeger et al. [39] also reported similar results, i.e., that the multiple-response format than the single-response format exhibited a better performance in discriminating test samples based on evoked emotions.

Although previous studies have demonstrated the effect of response format (esp., single *vs*. multiple) on food-evoked emotions [38,39], there still exists a couple of research questions (RQ) to be investigated. First, it is worth testing a “sole” effect of the response format (i.e., single *vs*. multiple) on food-evoked emotions under the “within-participants design”. As summarized in Table 1, Jaeger et al. [38] combined two treatment variables, i.e., question layouts (circular *vs*. list) and response formats (single *vs*. multiple) under the “between-participants design”. In other words, each participant was assigned to one of the four groups (i.e., circular layout and single response, circular layout and multiple response, list layout and single response, and list layout and multiple response). Using the “between-participants design” has a merit to eliminate a potential carry-over effect of the “within-participants design”, where each participant takes part in multiple treatment conditions [40,41,42]. However, it is important to control for various factors that can influence food-evoked emotions (e.g., demographics, personality traits, dietary habits, scale usage, etc.) to ensure that any differences observed between test groups can be attributed to the sole effect of response formats being tested. Although Jaeger et al. [38] carefully controlled for demographic profiles among the four groups to ensure that significant findings could be attributed to differences between CEQ variants, we cannot rule out potential influences of participant characteristics that were not controlled for under the “between-participants design”. In this way, using the “within-participants design” can help researchers generalize the effect of the response format (i.e., single *vs*. multiple) on food-evoked emotions measured using the CEQ or its variants. Therefore, this study aimed to determine whether the effect of response format on food-evoked emotions still exist under the “between-participants design” (RQ 1).

Another question worth investigating is whether the effect of response format on evoked emotions, as measured by the CEQ or its variants, is still present when responding to other types of food-related stimuli, especially food images (RQ 2). Although written names of food items were employed as test samples [38,39], images of food items have not yet been used. This presents an opportunity to gather additional evidence regarding the effect of response format on the emotions evoked by food-related stimuli. Written food names may allow individuals to imagine a wider spectrum of variety for each food item based on their previous experiences and contexts (e.g., “potato chips” have many variations in terms of appearance, ingredients, flavors, textures, and brands, and they are consumed in diverse contexts), yielding a broader range of emotional responses to each stimulus. On the other hand, food images may prompt individuals to imagine the same variety, resulting in more consistent responses to each stimulus among them. In other words, while written food names elicit learned associations between food items and emotions, food images may evoke emotions via both sensory (appearance) and cognitive factors [43]. Cardello et al. [43] observed that the magnitude of emotional responses differed between tasted food items and their corresponding names, although they still exhibited a strong correlation in the emotional profiles. Specifically, emotional food items (e.g., chocolates) evoked greater emotions when their names were presented compared to when they were tasted. However, food names that elicit low emotional responses (e.g., oatmeal or carrots) induced greater intensities of emotional attributes when the food items were tasted, which might be due to their sensory attributes [43]. Using the EsSense Profile^®^ [2], Piqueras-Fiszman and Jaeger [44] conducted a comparison of emotions between two treatment conditions: food images and tasted foods. Their study findings suggested that the profiles of emotional responses were generally similar between food images and tasted foods, although the degree of similarity varied depending on the type of food items. Because consumers responded similarly to both food images and actual foods [45,46,47], the use of food images as a substitute for tasted foods may be an option [44] when tasting samples is not feasible, such as during the COVID-19 pandemic or for online research purposes. In addition to the names of food items, written concepts of food items and real food samples were also used in the previous study regarding the effect of response format on food-evoked emotions measured using the CEQ or its variants [39]. However, no study has specifically examined the effect of response format on emotional responses to food stimuli using food images as test samples.

This research, comprised of Studies 1 and 2, aimed to determine the effect of response format by comparing emotional responses to “food images” between the two response formats: single *vs*. multiple under the “within-participants design”. Study 1 compared single and multiple response conditions with respect to food image-evoked emotions rated within “one remote session” of the within-participants design (i.e., each participant took part in both sessions within a session). To minimize both a potential carry-over effect of the “within-participants design” and an influence of environmental factors in the remote (online) testing, Study 2 did so over “two separated sessions” on two different days (i.e., one day for each response condition) at the individual sensory booths (with controlled ambient temperature and combined natural/artificial light) of the sensory evaluation facility.

## 2. Study 1—Comparison between Single and Multiple Response Conditions with Respect to Food Image-Evoked Emotions Rated within One Session in the Within-Participants Design

### 2.1. Materials and Methods

#### 2.1.1. Participants

Overall, 105 participants (57 females, 46 males, and 2 preferring not to say) of ages 19 to 74 years (mean age ± standard deviation (SD) = 44 ± 12 years) participated in this study. When conducting sensory studies using a CATA method for widely different samples, stable sample and descriptor configurations were obtained with 60–80 consumers [48]. Participants were recruited from Sejong University community (Seoul, Republic of Korea) and social networking services. All participants self-reported that they are Asians and were born in the Republic of Korea. The protocol used in this study was approved by the Institutional Review Board of Sejong University (SUIRB-HR-2022-012; SUIRB-HR-2023-002). Prior to their participation, the experimental procedure was explained, and an informed written consent was obtained from each participant.

#### 2.1.2. Food Images

Fourteen food images (Supplementary Figure S1) were purchased from a commercial web provider (Dreamstime, Brentwood, TN, USA), including images of balut (#132271516), birthday cake (#121006329), brewed coffee (#26448276; hereafter “coffee”), brewed green tea (#3972724; hereafter “green tea”), steamed and sliced broccoli (#238339622; hereafter “broccoli”), burnt toast bread slices (#47080332; hereafter “burnt toast”), cooked rice (#183353249), unpeeled boiled eggs and half eggs (#117215484; hereafter “boiled eggs”), grilled saury (#16175456; hereafter “grilled fishes”), Scottish haggis (#90784013; hereafter “haggis”), Kimchi (#24024491), molded oranges (#216627248), half rotten avocado (#96265489; hereafter “rotten avocado”), and beef steak (#34428041; hereafter “steak”). These food items, varying in valence, arousal, and familiarity, were selected based on previous studies related to food-evoked emotions [2,33,39,49,50] and food image database [51,52,53]. The sizes of individual image samples were edited to be similar (height × width = 970 × 680 pixels).

#### 2.1.3. Procedure

To minimize the risk of contracting and spreading COVID-19, volunteers in this study participated using their computerized devices such as smartphones, tablets, or laptops. Previous research has shown that intensity ratings did not differ for 19 of 20 sensory attributes among the three computerized device conditions: iPods, iPads, and laptops with external monitor displays [54]. Participants were asked to self-report their hunger/fullness level on a line scale ranging from −50 (“very hungry”) to 50 (“very full”). They self-reported their feelings in terms of valence on a line scale ranging from −50 (“very unpleasant”) to 50 (“very pleasant”). They also self-reported their feelings in terms of arousal on a line scale ranging from −50 (“very deactivated/low arousal”) to 50 (“very activated/high arousal”). On average, participants’ hunger/fullness and feeling of arousal were close to neutral, i.e., hunger/fullness (mean ± SD = 9.69 ± 23.55) and arousal (4.65 ± 21.31). They were likely to lean toward slightly pleasant in terms of valence (mean ± SD = 16.14 ± 19.72).

Participants were presented with 14 food image samples in sequential monadic fashion, randomized using a Williams Latin square design. Participants were asked to imagine that the food item shown in each image sample is in front of them and they are consuming it. They were then asked to select a pair of emotion terms dominantly representing their emotional responses to each image sample from the list of 12 pairs of emotion terms from the questionnaire CEQ [35], referred to as a “single response” (SR) condition. As shown in Figure 1, CEQ is composed of 12 pairs of emotion terms: “active/alert”, “energetic/excited”, “enthusiastic/inspired”, “happy/satisfied”, “secure/at ease”, “relaxed/calm”, “passive/quiet”, “dull/bored”, “blue/uninspired”, “unhappy/dissatisfied”, “tense/bothered”, and “jittery/nervous” [35]. For Korean participants, to minimize a potential gap between original (English) and translated versions of the emotion terms, both Korean and English versions of the 12 pairs of emotion terms were presented. The 12 pairs of emotion terms, with the assistance of bilingual individuals proficient in both English and Korean, were translated into Korean based on previous studies that utilized Korean versions of self-reported emotion questionnaires, including the EsSense Profile^®^ [55] and the Coffee Drinking Experience questionnaire [56]. Prior to the main test, clarity of the translated terms was also checked with Korean individuals. Moreover, since food-evoked emotions did not vary with the question layout, i.e., circular (e.g., Figure 1A) *vs*. conventional list layout [38], the twelve emotion terms were listed across three columns (i.e., three columns × four rows) as depicted in Figure 1B.

Following the SR condition, participants were again presented with the food image sample and asked to select all terms representing their evoked emotions from the list of 12 pairs of emotion terms, referred to as a “multiple response” (MR) condition. For both SR and MR conditions, to lead participants to pay attention to the list of emotion terms, the order of the 12 pairs of emotion terms was randomized across image samples and participants. There was no time limit when selecting emotion terms. Afterwards, participants were asked to rate their feelings in terms of valence and arousal on the line scales described above. Participants were allowed a minimum of three seconds between the sample presentations, allowing them to control the pace at which they evaluated the samples. After each break, participants were asked to focus on a black cross against a white background for two seconds to ensure they were ready to pay attention to upcoming food image samples. Experimental instructions, food image samples, and scales were presented using Compusense Cloud^®^ software (Compusense Inc., Guelph, ON, Canada).

#### 2.1.4. Data Analysis

Data analysis was conducted using XLSTAT software (Addinsoft, New York, NY, USA) and JMP Pro (version 16, SAS Institute Inc., Cary, NC, USA) software. Considering previous findings [38,39], the SR and MR conditions were compared with respect to four aspects. Using paired *t*-tests, we first tested whether the response conditions, i.e., SR *vs*. MR, could affect the frequency of citations per food image sample for each pair of emotion terms. Second, it was next determined whether the SR and MR conditions could differ in terms of a capacity to discriminate test samples based on evoked emotions. A Cochran’s *Q*-test was performed to test whether the 14 food image samples could differ in the proportions of selection by participants for each pair of emotion terms. If there was a significant difference, *post hoc* multiple pairwise comparisons were conducted using a critical difference (Sheskin) procedure. Subsequently, the proportions of significant pairwise sample comparisons, determined by *post hoc* multiple pairwise comparisons, among the 91 possible comparisons (i.e., (14 × 13)/2) across the 14 image samples were compared between the SR and MR conditions using a *z*-test. Third, associations between food image samples and pairs of emotion terms under either the SR or the MR condition were determined using correspondence analysis (CA). To identify whether the configurations of the biplots of the CAs drawn in the SR and MR conditions were different with respect to food images or emotion term pairs, regression vector (RV) coefficients were computed. The RV coefficients were considered to be a correlation coefficient in a multidimensional configuration [57,58]. Finally, the RV coefficients were employed to determine whether the configurations of the biplots of the CAs drawn in the SR and MR conditions were similar with the biplot configurations of the principal component analysis (PCA) based on the mean ratings of valence and arousal for the 14 food image samples. An analysis of variance (ANOVA) was also conducted to determine whether the 14 food image samples differed in terms of ratings of valence or arousal. If a significant difference was identified, *post hoc* multiple pairwise comparisons between the food image samples were conducted using Tukey’s Honestly Significant Difference (HSD) tests. A statistical significance was defined to exist when *p* < 0.05.

### 2.2. Results and Discussion

#### 2.2.1. Mean Frequency of Citations per Food Image Sample for Each Pair of Emotion Terms

**Figure 2:**
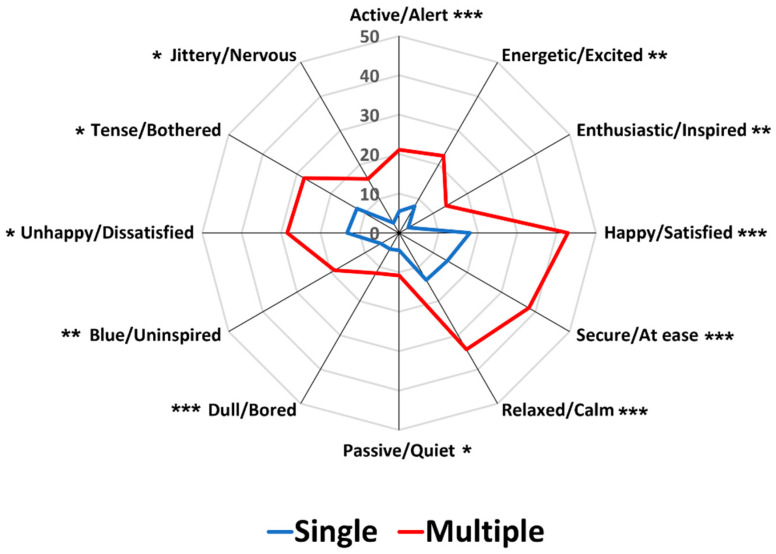
Comparisons between single and multiple response conditions with respect to mean frequency of citations per food image sample for each emotion term in Study 1. *, **, and *** represent a significant difference at *p* < 0.05, *p* < 0.01, and *p* < 0.001, respectively.

Figure 2 represents a comparison between single and multiple response conditions with respect to mean frequency of citations per food image sample for each pair of emotion terms. As expected, the MR condition exhibited significantly higher frequencies of citations than the SR condition for each food image (*p* < 0.05), in line with previous studies where the total frequency counts for each pair of emotion terms of the CEQ increased when the multiple response option was available [38,39]. Interestingly, the patterns of citation for each pair of emotion terms were almost identical between the SR and MR conditions, suggesting that the MR condition only boosts the frequency of citation obtained in the SR condition.

#### 2.2.2. Discriminability between Food Image Samples for Each Pair of Emotion Terms

Supplementary Tables S1 and S2 are contingency tables of the proportions of citations by participants across the 14 food image samples for each pair of emotion terms under the SR and MR conditions, respectively. While Cochran’s *Q*-tests revealed significant sample differences for all pairs of emotion terms (*p* < 0.001) (Supplementary Table S1), *post hoc* multiple pairwise comparisons using the critical difference (Sheskin) procedure found no significant pairwise sample comparisons for the “passive/quiet” emotion (*p* > 0.05). This lack of significant difference was most likely due to the low proportions of citations (i.e., ≤0.10) across all image samples for the pair of emotion terms. For the MR condition, Cochran’s *Q*-tests also found significant sample differences for all pairs of emotion terms (*p* < 0.001) (Supplementary Table S2). In contrast to the SR condition, the MR condition produced significant sample differences as determined by *post hoc* multiple pairwise comparisons with respect to the “passive/quiet” emotion (*p* < 0.05), mostly because of a higher proportion of citation (i.e., 0.31) for the green tea image (Supplementary Table S2).

**Figure 3:**
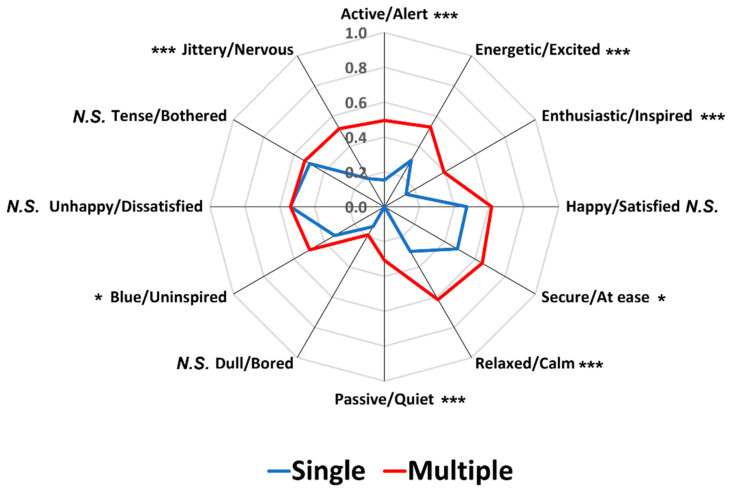
Comparisons between single and multiple response conditions with respect to the proportion of significant pairwise sample comparisons among the 91 possible comparisons across the 14 food image samples for each pair of emotion terms in Study 1. * and *** represent a significant difference at *p* < 0.05 and *p* < 0.001, respectively. *N.S.* represents no significant difference at *p* < 0.05.

Figure 3 illustrates comparisons between the SR and MR conditions in terms of the proportion of significant pairwise sample comparisons among the 91 possible comparisons across the 14 image samples for each pair of emotion terms. While the two conditions did not differ in the four pairs of emotion terms—“happy/satisfied” (*p* = 0.07), “dull/bored” (*p* = 0.42), “unhappy/dissatisfied” (*p* = 1.00), and “tense/bothered” (*p* = 0.77), the MR condition produced higher proportions of the significant pairwise sample comparisons than the SR condition in the eight emotion terms (*p* < 0.05). To some extent, these results support previous findings that MR conditions exhibited a higher ability of sample discrimination based on food-evoked emotions than SR conditions [38,39]. Notably, in the previous study [38], the percentages of significant pairwise sample comparisons were different between the SR and MR conditions, especially when the emotion terms were presented in an original circular layout, but not when presented in a conventional layout format. However, the present study using the conventional layout format demonstrated that the MR condition has a higher discriminability of pairwise sample comparisons than the SR condition. Thus, a further conclusive study is needed to test whether a significant interaction between layout variants and response formats exists in the effect of response condition on the discriminability of test samples based on food-evoked emotions. Another point to note is that significant differences between the SR and MR conditions were observed, especially in the pairs of emotion terms placed in the space of positive valence (Figure 3), suggesting that the sample differences in evoked emotions depend on the affective characteristics of test samples, but not on the variant types of response condition (also see [39]).

#### 2.2.3. Associations between Food Image Samples and Pairs of Emotion Terms

**Figure 4:**
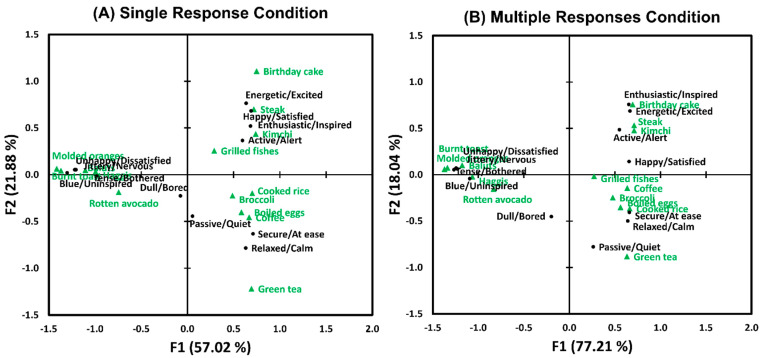
Biplots of the correspondence analyses showing the associations between the 14 food image samples (green triangles) and the 12 pairs of emotion terms (black circles) in the single (**A**) and multiple (**B**) response conditions in Study 1.

As shown in Figure 4, biplots of CAs represent associations between the 14 food image samples (green triangles) and the 12 pairs of emotion terms (black circles) for single (A) and multiple (B) response conditions, respectively. The biplots of the SR and MR conditions exhibited both similar and different patterns in terms of associations between food image samples and their evoked emotions. More specifically, while the total variances of the associations between food image samples and evoked emotions were mainly explained by the *X*-axis (F1, “valence”-related) under both SR and MR conditions, the total variances accounted for by the *X*-axis in the MR condition (72.21%) were higher than those for the SR condition (57.02%). This result suggests that participants used the pairs of emotion terms related to the “valence” dimension more often than those related to the “arousal” dimension, which is in line with previous studies demonstrating that test samples were differentiated by emotional responses, mainly their positive/negative valence [59,60]. It is worth noting that while the food image samples located on the positive side of the *X*-axis (F1, “valence”-related) were separated over a wider range of the *Y*-axis (F2, “arousal”-related), those located on the negative side of the *X*-axis were distributed within a narrower range of the *Y*-axis. This result is in line with a previous study asserting that activation (arousal) can be the main discriminating factor in positive emotions [61]. On the other hand, since these observations also suggest that the food image samples used in this study did not elicit a wide range of arousal levels when rated with negative valence dimension, a further study using test samples with a wider range of arousal levels should be conducted to confirm the interpretation of this result.

The configurations of the two CA biplots drawn for the SR and MR conditions, respectively, were found to be similar with respect to both food images (RV coefficient = 0.97, *p* < 0.001) and emotional responses (RV coefficient = 0.96, *p* < 0.001) [57,58] (Table 2). In other words, the configurations of the associations between the food image samples and pairs of emotion terms did not differ as a function of response format, in congruence with previous findings [38,39]. This result, similarity between the configurations of the two CA biplots drawn in the SR and MR conditions, is also consistent with observations of the SR and MR conditions that exhibit almost identical patterns with respect to the frequency of citation per food image sample for each pair of emotion terms (Figure 2).

#### 2.2.4. Similarities to the Configurations of Valence and Arousal Dimensions in Response to Food Image Samples

**Figure 5:**
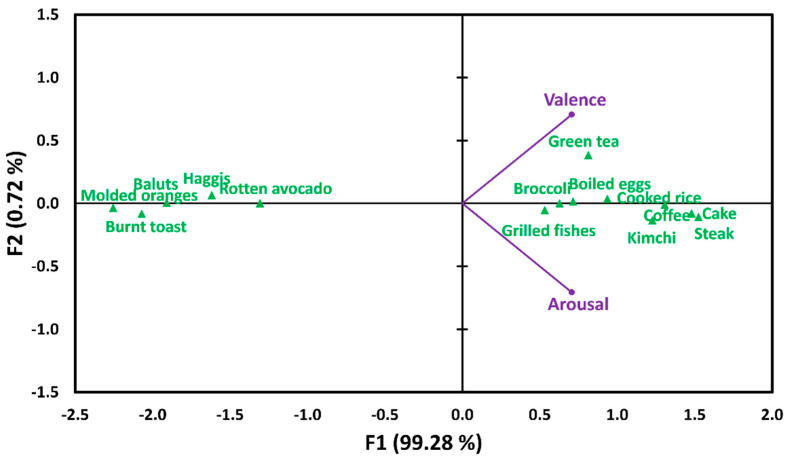
A biplot of the principal component analysis based on the mean ratings of valence and arousal for the 14 food image samples (green triangles).

The 14 food image samples were found to differ significantly in terms of mean ratings of valence (*F* = 264.74, *p* < 0.001) or arousal (*F* = 121.05, *p* < 0.001). Since identification of food image samples with higher ratings of valence or arousal was not the main objective of this study, the results of *post hoc* multiple pairwise comparisons with respect to valence or arousal are presented in Supplementary Table S3. Figure 5 represents a biplot of PCA based on mean ratings of valence and arousal for the 14 food image samples, accounting for 100.00% of total variance. Specifically, 99.28% of total variance was accounted for by *X*-axis (F1) and the two dimensions of valence and arousal were highly correlated (*r*(14) = 0.986, *p* < 0.001).

The RV coefficients revealed that the configurations of the CA biplot obtained from the association between food image samples and pairs of emotion terms in the SR and MR conditions were found to be similar with those of the PCA biplot based on mean ratings of valence and arousal for the food image samples [57,58]. The MR condition exhibited a slightly higher RV coefficient (0.96, *p* < 0.001) than the SR condition (0.92, *p* < 0.001) (Table 3), indicating that the association between the food image samples and pairs of emotion terms in the CEQ is likely to be better matched to the valence and arousal dimensions of the food images under the MR condition than under the SR condition.

Taken together, Study 1 showed that both SR and MR conditions were effective in characterizing emotional responses to food image samples, confirming RQs 1 and 2. While both conditions exhibited similar result patterns, the MR condition exhibited better performance, especially in the discrimination of food image samples based on evoked emotions and the similarity to configurations of valence and arousal dimensions for the test samples. However, since the SR and MR conditions in Study 1 were conducted remotely within a session, to further substantiate the results of Study 1, Study 2 was conducted to test the effect of the response conditions at individual sensory booths in separated sessions over two different days.

## 3. Study 2—Comparison between Single and Multiple Response Conditions with Respect to Food Image-Evoked Emotions Rated over Separated Sessions of the Within-Participants Design

### 3.1. Materials and Methods

#### 3.1.1. Participants

Overall, 64 participants (43 females, 20 males, and 1 preferring not to say) ranging from age 20 to 82 years (mean age ± SD = 44 ± 17 years) participated in this study. Participants were recruited from the Northwest Arkansas community through the University of Arkansas Sensory Science Center (Fayetteville, AR, USA). All participants self-reported no clinical history of major diseases and a normal sense of vision. Supplementary Table S4 lists the participants’ demographic profiles. The protocol used in this study (2110361492) was approved by the Institutional Review Board of the University of Arkansas (Fayetteville, AR, USA). Prior to participation, the experimental procedure was explained, and an informed written consent was obtained from each participant.

#### 3.1.2. Food Images

The 14 food images (Supplementary Figure S1) of Study 1 were used.

#### 3.1.3. Procedure

Each volunteer participated in this study twice, once under the SR condition and the other under the MR condition, on two different days (three days apart), with the order of the SR and MR conditions randomized across participants. Participants were asked to refrain from eating, cigarette smoking, and drinking (except for drinking water) for 2 h prior to participation [62]. To avoid potential influences of environmental contexts on consumer responses to food image samples, participants were asked to evaluate the test samples in the individual sensory booths at the University of Arkansas Sensory Science Center. Although there was a minimal effect of the computerized devices with respect to responses to test samples [54], experimental instructions, food image samples, and scales were presented using Compusense Cloud^®^ software, on the identically sized (17-inches) external monitors of desktop PCs. As in Study 1, participants self-reported their hunger/fullness level and their feelings of valence and arousal on the line scales ranging from −50 to 50. On average, participants’ hunger/fullness level (mean ± SD = −3.73 ± 25.24) was close to neutral, i.e., neither full nor hungry. Overall, they were likely to be slightly activated (11.33 ± 23.51) and pleasant (mean ± SD = 24.50 ± 21.69).

Procedures for sample presentation and evaluation were identical to those in Study 1. As mentioned above, unlike in Study 1, each participant took part in two sessions on different days (three days apart) for SR and MR conditions, respectively.

#### 3.1.4. Data Analysis

Data analysis was also conducted in the same manner as in Study 1.

### 3.2. Results and Discussion

#### 3.2.1. Mean Frequency of Citations per Food Image Sample for Each Pair of Emotion Terms

**Figure 6:**
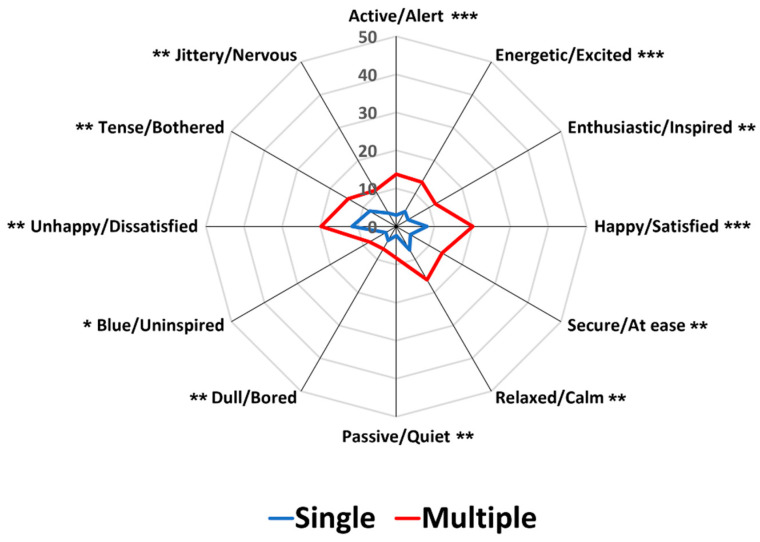
Comparisons between single and multiple response conditions with respect to mean frequency of citations per food image sample for each emotion term in Study 2. *, **, and *** represent a significant difference at *p* < 0.05, *p* < 0.01, and *p* < 0.001, respectively.

As shown in Figure 6, the MR condition, when compared to the SR condition, showed significantly higher frequencies of citations per food image sample for each pair of emotion terms (*p* < 0.05), supporting the result of Study 1 (Figure 2) and previous findings [38,39]. As can be observed in Figure 2 of Study 1, the patterns of citation for each pair of emotion terms were similar between SR and MR conditions in Study 2 as well, although both conditions were tested over two different days.

#### 3.2.2. Discriminability between Food Image Samples for Each Pair of Emotion Terms

Supplementary Tables S5 and S6 are contingency tables of the proportions of citations by participants across the 14 food image samples for each pair of emotion terms under the SR and MR conditions, respectively. Cochran’s *Q*-tests found significant sample differences for individual pairs of emotion terms (for all, *p* < 0.001), except for the “blue/uninspired” emotion (*p* = 0.07) for the SR condition (Supplementary Table S5). A lack of significance for the “blue/uninspired” emotion was most likely due to the low proportions of citations (i.e., ≤0.11) across all image samples. Cochran’s *Q*-tests also found significant sample differences for all pairs of emotion terms (for all, *p* < 0.001) under the MR condition (Supplementary Table S6).

**Figure 7:**
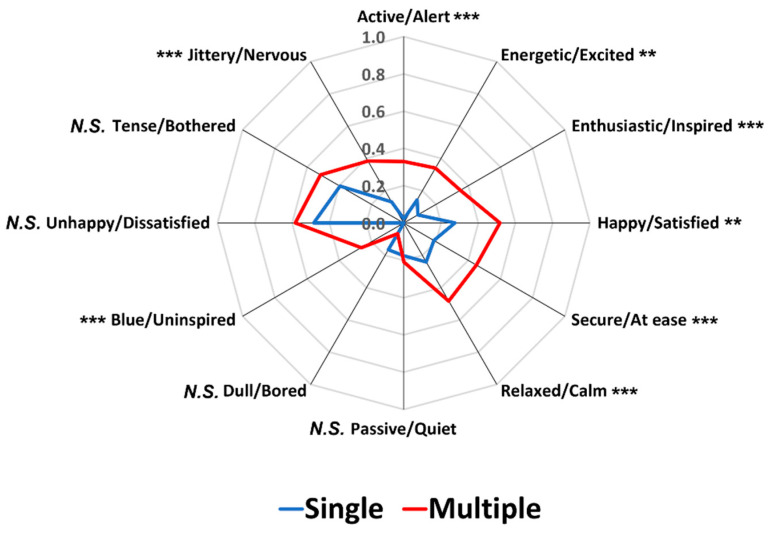
Comparisons between single and multiple response conditions with respect to the proportion of significant pairwise sample comparisons among the 91 possible comparisons across the 14 food image samples for each pair of emotion terms in Study 2. ** and *** represent a significant difference at *p* < 0.01 and *p* < 0.001, respectively. *N.S.* represents no significant difference at *p* < 0.05.

Figure 7 represents comparisons between the SR and MR conditions with respect to the proportion of significant pairwise sample comparisons among the 91 possible combinations across the 14 food image samples for each pair of emotion terms. The SR and MR conditions did not differ with respect to the four pairs of emotion terms: “passive/quiet” (*p* = 0.71), “dull/bored” (*p* = 0.06), “unhappy/dissatisfied” (*p* = 0.23), and “tense/bothered” (*p* = 0.13), similar to the result of Study 1 that showed no difference with respect to “happy/satisfied”, “dull/bored”, “unhappy/dissatisfied”, and “tense/bothered” emotions. However, the MR condition exhibited higher proportions than the SR condition for the significant pairwise sample comparisons in the eight emotion terms (*p* < 0.05), concurring with the results of Study 1 and previous findings [38,39].

#### 3.2.3. Associations between Food Image Samples and Pairs of Emotion Terms

**Figure 8:**
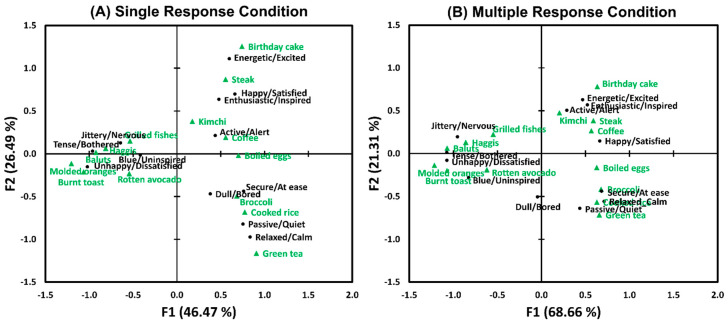
Biplots of the correspondence analyses showing the associations between the 14 food image samples (green triangles) and the 12 pairs of emotion terms (black circles) in the single (**A**) and multiple (**B**) response conditions in Study 2.

As shown in Figure 8, the biplots of the SR and MR conditions displayed similar patterns in the associations between the 14 food image samples and the 12 pairs of emotion terms. As in Study 1, the *X*-axis (F1, “valance-related”) accounted for greater percentages of the total variances under both SR and MR conditions, and the total variances explained by the *X*-axis in the MR condition (68.66%) were greater than those under the SR condition (46.47%). In addition, the first two axes (i.e., F1 and F2) of CA in the MR condition (89.97%) accounted for the total variance more than those in the SR condition (72.96%).

The configurations of the two CA biplots obtained under the SR and MR conditions, respectively, were similar in terms of food images (RV coefficient = 0.95, *p* < 0.001) or emotional responses (RV coefficient = 0.88, *p* < 0.001) (Table 2). This result agrees with the results of Study 1 and previous finding [38].

#### 3.2.4. Similarities to the Configurations of Valence and Arousal Dimensions in Response to Food Image Samples

The configurations of the CA biplot drawn from the association between food image samples and pairs of emotion terms in the SR and MR conditions, respectively, were similar with those of the PCA biplot based on mean ratings of valence and arousal for the food image samples. As in Study 1, the MR condition reflected a higher RV coefficient (0.84, *p* < 0.001) than the SR condition (0.75, *p* < 0.001) (Table 3). In other words, the association between the food image samples and pairs of the emotion terms in the CEQ was likely to be better matched to the valence and arousal dimensions of the food images under the MR condition than under the SR condition.

## 4. General Discussion

This study provided strong evidence to support previous findings that (1) both SR and MR conditions are useful in determining profiles of emotional responses to test samples and (2) the MR condition has a greater capacity to differentiate test samples based on the emotions they elicit. Regarding the research questions at hand, the results of this study demonstrated that the effect of response format (i.e., SR *vs*. MR) on emotional responses to food-related stimuli remains under the “between-participants design” (RQ 1) and when utilizing food images as test samples (RQ 2). This study also revealed new insights from four perspectives, as follows:

First, the MR condition resulted in a higher frequency of citations per food image sample for each pair of emotion terms than the SR condition. A study by Jaeger et al. [38] found that the MR condition significantly increased the total frequency of citations for 8 pairs of emotion terms, but not for 4 of the 12 pairs of emotion terms: “unhappy/dissatisfied”, “dull/bored”, “tense/bothered”, and “jittery/nervous”. However, in both Studies 1 and 2 of this study, the MR condition exhibited a higher frequency of citation per sample for all pairs of emotion terms than the SR condition (Figure 2 and Figure 6). This finding underlines the importance of recognizing that food samples (food images in this study) can elicit more than one pair of emotions, highlighting the need to employ a set of multiple emotion terms and a multiple response format when measuring food-evoked emotions. Self-reported emotion questionnaires published in peer-reviewed journals consist of multiple emotion terms [2,35], and most questionnaires use the scales for rating intensities of individual emotion attributes [2]. However, because ratings on scales demand time and cognitive load, a CATA-based variant has also been applied for resolving practical challenges. For example, while the EsSense Profile^®^ is comprised of rating scales, researchers often use its CATA-based variant format [34]. Therefore, multiple response formats such as CATA or Rate-All-That-Apply (RATA) may facilitate individual responses to the CEQ because they do not require spending time and effort in selecting only one [34,63]. However, it should also be noted that the patterns of citations for each pair of emotion terms under the MR condition were almost identical with those under the SR condition, as observed in Studies 1 and 2. This finding suggests that the SR condition is still effective when identifying patterns of emotion profiles.

Second, the MR condition exhibited a higher capacity to differentiate test samples based on evoked emotions than the SR condition. The MR condition differentiated the 14 food image samples based on the proportions of citations for the 12 pairs of emotion terms in both Studies 1 and 2. However, the SR condition did not differentiate the test samples in one of the 12 pairs of emotion term in either study because of the low proportions of citations across the 14 image samples in the SR condition. The MR conditions also exhibited higher proportions of significant pairwise sample comparisons than the SR conditions in 8 out of 12 pairs of emotion terms in both Studies 1 and 2. Notably, significant differences between the SR and MR conditions were observed in the pairs of emotion terms related to the positive valence dimension, suggesting that participants focus more on emotion terms of positive valence when the multiple response option was available. In fact, among emotions experienced in the contexts of food consumption, positive valence-related emotions (e.g., satisfaction, enjoyment, and desire) occurred more often than negative valence-related emotions (e.g., sadness, anger, and jealousy) [64,65,66]. However, when a single response option was provided, participants tended to focus more on emotion terms of negative valance, resulting in no significant difference between the SR and MR conditions in those pairs of emotion terms (Figure 3 and Figure 7). Previous studies regarding the structure of emotions in general included negative emotion terms more than positive emotion terms [61,67], and individuals tend to remember negative emotions or experiences more than positive ones. Thus, when participants were asked to select one pair of emotion terms only, they were more likely to characterize their emotional responses to food image samples using negative pairs of emotion terms.

Third, in both Studies 1 and 2, the first two dimensions of the CA biplots accounted for more than 70% of the total variances in the associations between food image samples and evoked emotions. In addition, pairs of emotion terms under the MR condition accounted for more portion of the total variances than those under the SR condition (Figure 4 and Figure 8), which is linked to the results showing the MR condition’s higher discriminability on food image samples based on their evoked emotions (Figure 3 and Figure 7). It is also worth noting that the configurations of the two CA biplots drawn for the SR and MR conditions were similar (Table 2). This supports the previous finding that showed a similarity between the SR and MR conditions in terms of the configuration of the CA biplots of the association between written food names and pairs of emotion terms [38]. This result also suggests that the SR condition can serve as a viable alternative for characterizing emotional response profiles of test samples when the MR condition is not feasible due to experimental design (e.g., when there are many test samples with time limitations) or participant-related factors (e.g., when participants have a difficulty in maintaining attention during lengthy tasks) [38].

Fourth, in both Studies 1 and 2, the MR condition was more likely than the SR condition to match the configurations of the food image samples with the configurations based on valence and arousal ratings of the image samples (Table 3). This result suggests that the MR condition more than the SR condition may enhance effectiveness of the CEQ for characterizing test samples based on evoked emotions based on valence and arousal dimensions. However, this does not mean that researchers and professionals should use the MR condition instead of the SR condition, because the SR condition’s CA biplot configurations were also found to be highly correlated to the PCA biplot configurations drawn by the ratings of valence and arousal for food image samples.

While this study has contributed new findings and insights to previously unexplored areas, there are still several aspects that require further exploration. Although food image samples were used as test samples in order to mitigate participants’ sensory fatigue and minimize the risk of COVID-19 transmission during the current pandemic, conducting a future study using real food samples within diverse contexts of food experience [44] would enhance the robustness of the current findings. Previous studies have shown differences between food images and real food items in terms of evoked emotion profile [43,47]. In addition, even though food image samples varying in valence, arousal, and familiarity were chosen based on previous studies, their arousal levels were likely to fall into a narrower range than expected. Hence, further research encompassing a broader spectrum of valence and arousal levels would contribute to generalizing the current findings. Furthermore, although this study was not specifically designed for a cross-cultural comparison between two different countries, the results indicate a similar observation of the effect of response format on emotional responses to food images in both countries. It would be interesting to explore whether cultural backgrounds influence the effect of response conditions on food-evoked emotions by employing the same experimental setup in different countries [35].

## 5. Conclusions

In conclusion, this study, conducted under a “between-participants design”, supports previous findings [38,39] and provides robust empirical evidence that both single and multiple response conditions in the CEQ variant effectively characterize profiles of emotional responses to food samples, particularly food image samples in this study. Both response conditions exhibited similar patterns of results. However, our findings suggest that the MR condition, when compared to the SR condition, may be more advantageous in differentiating test samples based on their evoked emotions or projecting food-evoked emotions onto the dimensions of valence and arousal in response to the test samples.

The findings of this research have significant implications for professionals in the food industry and researchers in the field of sensory and consumer sciences by shedding light on the impact of response format on emotions evoked by food images within the context of the CEQ variant. Specifically, the original response format (i.e., single response) of the CEQ or its variants should be taken into account when there are time constraints and many samples to be tested, when participants are likely to struggle to maintain attention during lengthy tasks, when clear differentiation in evoked emotions among samples is expected to exist, or only when profiling emotional responses to test samples (i.e., without sample comparisons). However, the multiple response format should be considered when test samples are expected to exhibit a small difference in food-evoked emotions or when the goal is to thoroughly compare test samples in terms of evoked emotions. Taken together, these findings will facilitate the practical application and interpretation of the CEQ and its variants in diverse domains.

## Figures and Tables

**Table 1:** A summary of previous and present studies that investigated the effect of variants in the valence × arousal circumplex-inspired emotion questionnaire (CEQ) with respect to experimental condition.

Reference	Study	CEQ Variant ^1^	Sample Type	Sample Module	Test Type	Test Place	Experimental Design	No. of Participants
Jaeger et al. [38]	1	C and S *vs*. C and M *vs*. L and S *vs*. L and M	15 foods	Written names	Remote	New Zealand	Between-participants	160 *vs*. 160 *vs*. 160 *vs*. 160
Jaeger et al. [39]	1	C and S *vs*. L and M	9 fruits	Tasted	On site	New Zealand	Between-participants	91 *vs*. 94
	2	C and S *vs*. L and M	9 candies	Tasted	On site	New Zealand	Between-participants	91 *vs*. 94
	3	C and S *vs*. L and M	5 kiwifruits	Written concepts	Remote	UK	Between-participants	209 *vs*. 210
	4	C and S *vs*. C and M	17 foods	Written names	Remote	USA	Between-participants	628 *vs*. 625
The present study	1	S *vs*. M	14 foods	Pictures	Remote	Republic of Korea	Within-participants	105
	2	S *vs*. M	14 foods	Pictures	On site	USA	Within-participants	64

^1^ C = circular layout; L = list layout; S = single response; M = multiple response.

**Table 2:** RV coefficients showing the similarity between the single and multiple response conditions with respect to the configurations of the correspondence analysis biplots based on the associations between the 14 food image samples and the 12 pairs of emotion terms in Studies 1 and 2.

Study No.	Category	RV Coefficient	*p*-Value
Study 1	Food image samples	0.97	<0.001
	Pairs of emotion terms	0.96	<0.001
Study 2	Food image samples	0.95	<0.001
	Pairs of emotion terms	0.88	<0.001

**Table 3:** RV coefficients showing the similarity between the biplot configurations of the correspondence analysis (CA) and principal component analysis (PCA) with respect to the projection of food image samples in Studies 1 and 2.

Study No.	Response Condition	RV Coefficient	*p*-Value
Study 1	Single response	0.92	<0.001
	Multiple response	0.96	<0.001
Study 2	Single response	0.75	<0.001
	Multiple response	0.84	<0.001

## Data Availability

The data are not publicly available due to the Institutional Review Board protocol guideline.

## Supplementary Materials

The following supporting information can be downloaded at: https://www.mdpi.com/article/10.3390/foods12112250/s1, Figure S1: Fourteen food image samples used in this study; Table S1: A contingency table of the proportions of citations by 105 participants across the 14 food image samples for individual pairs of emotion terms in the single response condition of Study 1; Table S2: A contingency table of the proportions of citations by 105 participants across the 14 food image samples for individual pairs of emotion terms in the multiple response condition of Study 1; Table S3: Mean comparisons among the 14 food image samples with respect to valence or arousal dimension; Table S4: Demographic profile of the 64 participants in Study 2; Table S5: A contingency table of the proportions of citations by 64 participants across the 14 food image samples for individual pairs of emotion terms in the single response conditions of Study 2; Table S6: A contingency table of the proportions of citations by 64 participants across the 14 food image samples for individual pairs of emotion terms in the multiple response conditions of Study 2.
