# Supplementary material for: The Effect of Response Conditions on Food Images-Evoked Emotions Measured Using the Valence × Arousal Circumplex-Inspired Emotion Questionnaire (CEQ)
Source: Foods. 2023 Jun 2;12(11):2250. doi: 10.3390/foods12112250 (PMC10252207; doi:10.3390/foods12112250)
Supplement: Supplementary file 1 [file foods-12-02250-s001.zip › foods-2344020-supplementary.pdf]

Supplementary Table S1. A contingency table of the proportions of citations by 105 participants across the 14 food image samples for individual pairs of emotion terms in the single response condition of Study 1

Emotion-related terms	BA[1]	BE	BR	BT	CA	CO	CR	GT	GF	HA	KI	MO	SA	ST	*p*-value
Active/<br>Alert	0.01[2]<br>c	0.02<br>c	0.10<br>abc	0.00<br>c	0.08<br>abc	0.13<br>ab	0.03<br>bc	0.00<br>c	0.07<br>abc	0.04<br>bc	0.16<br>a	0.00<br>c	0.01<br>c	0.10<br>abc	< 0.001
Energetic/<br>Excited	0.03<br>cde	0.04<br>cde	0.10<br>bcde	0.00<br>e	0.20<br>ab	0.05<br>cde	0.07<br>cde	0.00<br>e	0.14<br>abcd	0.02<br>de	0.15<br>abc	0.00<br>e	0.01<br>e	0.25<br>a	< 0.001
Enthusiastic/<br>Inspired	0.00<br>b	0.03<br>b	0.04<br>b	0.00<br>b	0.02<br>b	0.02<br>b	0.00<br>b	0.00<br>b	0.05<br>b	0.01<br>b	0.15<br>a	0.00<br>b	0.00<br>b	0.04<br>b	< 0.001
Happy/<br>Satisfied	0.03<br>de	0.20<br>cd	0.12<br>cde	0.01<br>e	0.64<br>a	0.15<br>cde	0.30<br>bc	0.03<br>de	0.23<br>bc	0.02<br>e	0.24<br>bc	0.00<br>e	0.04<br>de	0.40<br>b	< 0.001
Secure/<br>At ease	0.01<br>fg	0.31<br>abc	0.23<br>bcd	0.00<br>g	0.01<br>fg	0.17<br>cdef	0.43<br>a	0.36<br>ab	0.11<br>defg	0.00<br>g	0.20<br>bcde	0.00<br>g	0.02<br>fg	0.04<br>efg	< 0.001
Relaxed/<br>Calm	0.02<br>bc	0.18<br>b	0.16<br>bc	0.00<br>c	0.04<br>bc	0.41<br>a	0.09<br>bc	0.52<br>a	0.10<br>bc	0.01<br>c	0.07<br>bc	0.00<br>c	0.10<br>bc	0.15<br>bc	< 0.001
Passive/<br>Quiet	0.04<br>a	0.08<br>a	0.09<br>a	0.01<br>a	0.00<br>a	0.03<br>a	0.07<br>a	0.06<br>a	0.05<br>a	0.07<br>a	0.02<br>a	0.00<br>a	0.10<br>a	0.00<br>a	< 0.001
Dull/<br>Bored	0.05<br>abc	0.12<br>a	0.11<br>ab	0.02<br>bc	0.01<br>bc	0.01<br>bc	0.01<br>bc	0.02<br>bc	0.10<br>abc	0.08<br>abc	0.00<br>c	0.00<br>c	0.11<br>ab	0.01<br>bc	< 0.001
Blue/<br>Uninspired	0.06<br>abc	0.01<br>c	0.05<br>bc	0.13<br>ab	0.00<br>c	0.00<br>c	0.01<br>c	0.00<br>c	0.05<br>bc	0.13<br>ab	0.00<br>c	0.12<br>ab	0.15<br>a	0.01<br>c	< 0.001
Unhappy/<br>Dissatisfied	0.34<br>b	0.01<br>c	0.01<br>c	0.52<br>a	0.00<br>c	0.00<br>c	0.00<br>c	0.01<br>c	0.05<br>c	0.22<br>b	0.00<br>c	0.36<br>b	0.24<br>b	0.00<br>c	< 0.001
Tense/<br>Bothered	0.36<br>a	0.00<br>d	0.01<br>d	0.26<br>ab	0.01<br>d	0.03<br>cd	0.01<br>d	0.00<br>d	0.05<br>cd	0.32<br>ab	0.01<br>d	0.40<br>a	0.18<br>bc	0.10<br>d	< 0.001
Jittery/<br>Nervous	0.06<br>abc	0.00<br>c	0.00<br>c	0.05<br>abc	0.00<br>c	0.00<br>c	0.00<br>c	0.00<br>c	0.03<br>bc	0.09<br>ab	0.00<br>c	0.11<br>a	0.06<br>abc	0.00<br>c	< 0.001

[1]BA = baluts; BE = boiled eggs; BR = broccoli; BT = burnt toast; CA = cake; CO = coffee; CR = cooked rice; GT = green tea; HA = haggis; KI = Kimchi; MO = molded oranges; RA = rotten avocado; ST = steak

[2]The proportions with different letters within a row represent a significant difference at $p < 0.05$.

Supplementary Table S2. A contingency table of the proportions of citations by 105 participants across the 14 food image samples for individual pairs of emotion terms in the multiple response condition of Study 1

Emotion-related terms	BA[1]	BE	BR	BT	CA	CO	CR	GT	GF	HA	KI	MO	SA	ST	*P*-value
Active/ Alert	0.07[2] de	0.20 cde	0.31 bc	0.02 e	0.51 a	0.31 bc	0.10 de	0.04 de	0.22 cd	0.09 de	0.47 ab	0.02 e	0.06 de	0.41 ab	< 0.001
Energetic/ Excited	0.05 bcde	0.20 bcd	0.22 bc	0.01 de	0.68 a	0.24 b	0.20 bcd	0.00 e	0.22 bc	0.04 cde	0.50 a	0.00 e	0.03 cde	0.64 a	< 0.001
Enthusiastic/ Inspired	0.02 bc	0.09 bc	0.08 bc	0.01 c	0.37 a	0.17 b	0.09 bc	0.00 c	0.15 bc	0.03 bc	0.39 a	0.00 c	0.04 bc	0.41 a	< 0.001
Happy/ Satisfied	0.08 d	0.51 c	0.48 c	0.01 d	0.91 a	0.58 bc	0.64 bc	0.51 c	0.45 c	0.02 d	0.67 bc	0.00 d	0.11 d	0.77 ab	< 0.001
Secure/ At ease	0.02 h	0.61 bc	0.48 cd	0.01 h	0.25 efg	0.65 abc	0.82 ab	0.87 a	0.31 def	0.03 gh	0.51 cd	0.00 h	0.11 fgh	0.43 cde	< 0.001
Relaxed/ Calm	0.02 g	0.53 bcd	0.55 bc	0.00 g	0.18 efg	0.71 ab	0.53 bcd	0.90 a	0.27 ef	0.03 g	0.31 def	0.00 g	0.12 fg	0.40 cde	< 0.001
Passive/ Quiet	0.02 c	0.21 ab	0.18 ab	0.01 c	0.01 c	0.10 bc	0.12 bc	0.31 a	0.19 ab	0.11 bc	0.02 c	0.03 c	0.13 bc	0.00 c	< 0.001
Dull/ Bored	0.11 abcd	0.19 abc	0.22 a	0.09 abcd	0.01 d	0.05 cd	0.11 abcd	0.14 abcd	0.14 abcd	0.20 ab	0.02 d	0.07 bcd	0.23 a	0.01 d	< 0.001
Blue/ Uninspired	0.41 a	0.03 b	0.11 b	0.40 a	0.00 b	0.02 b	0.01 b	0.04 b	0.11 b	0.39 a	0.00 b	0.51 a	0.47 a	0.03 b	< 0.001
Unhappy/ Dissatisfied	0.73 ab	0.03 c	0.07 c	0.82 a	0.03 c	0.01 c	0.01 c	0.00 c	0.11 c	0.57 b	0.00 c	0.87 a	0.54 b	0.01 c	< 0.001
Tense/ Bothered	0.71 a	0.04 c	0.02 c	0.79 a	0.02 c	0.06 c	0.03 c	0.00 c	0.11 c	0.61 ab	0.03 c	0.82 a	0.46 b	0.02 c	< 0.001
Jittery/ Nervous	0.48 a	0.01 c	0.01 c	0.36 ab	0.02 c	0.03 c	0.00 c	0.00 c	0.09 c	0.40 ab	0.01 c	0.46 a	0.26 b	0.00 c	< 0.001

[1]BA = baluts; BE = boiled eggs; BR = broccoli; BT = burnt toast; CA = cake; CO = coffee; CR = cooked rice; GT = green tea; HA = haggis; KI = Kimchi; MO = molded oranges; RA = rotten avocado; ST = steak

[2]The proportions with different letters within a row represent a significant difference at $p < 0.05$.

Supplementary Table S3. Mean comparisons among the 14 food image samples with respect to valence or arousal dimension

Emotion-related terms	BA	BE	BR	BT	CA	CO	CR	GT	GF	HA	KI	MO	RA	ST	*P*-value
Valence	-29.63 ab	19.16 def	17.28 de	-34.26 a	31.59 h	29.67 gh	23.62 efg	27.82 gh	14.49 d	-23.13 bc	25.87 fgh	-36.80 a	-18.56 c	31.89 h	< 0.001
Arousal	-23.81 ab	14.86 cd	13.79 c	-24.89 a	27.58 f	24.00 def	17.85 cde	10.87 c	13.19 c	-20.41 ab	24.66 ef	-28.33 a	-14.84 b	28.69 f	< 0.001

BA = baluts; BE = boiled eggs; BR = broccoli; BT = burnt toast; CA = cake; CO = coffee; CR = cooked rice; GT = green tea; HA = haggis; KI = Kimchi; MO = molded oranges; RA = rotten avocado; ST = steak

[2]Mean ratings with different letters within a row represent a significant difference at $p < 0.05$.

Supplementary Table S4. Demographic profiles of the 64 participants in Study 2

Category	Subcategory	Frequency	Percentage (%)
Gender	Female	43	67.2
	Male	20	31.3
	Prefer not to answer	1	1.6
Age group	20 to 29 years old	19	29.7
	30 to 39 years old	10	15.6
	40 to 49 years old	11	17.2
	50 to 59 years old	11	17.2
	60 to 69 years old	7	10.9
	≥ 70 years old	6	9.4
Education	High school	8	12.5
	Some college	7	10.9
	2-year or 4-year college	24	37.5
	Master’s degree	19	29.7
	Doctoral or professional degree	6	9.4
Household income	< $20,000	16	25.0
	$20,000 to $39,999	13	20.3
	$40,000 to $59,999	8	12.5
	$60,000 to $79,999	10	15.6
	$80,000 to $99,999	8	12.5
	≥ $100,000	9	14.1
Ethnicity	Caucasian	45	70.3
	African American	7	10.9
	Asian	8	12.5
	Pacific Island	1	1.6
	Other	3	4.7

Supplementary Table S5. A contingency table of the proportions of citations by 64 participants across the 14 food image samples for individual pairs of emotion terms in the single response condition of Study 2

Emotion-related terms	BA	BE	BR	BT	CA	CO	CR	GT	GF	HA	KI	MO	SA	ST	*P*-value
Active/<br>Alert	0.03<br>ab	0.06<br>ab	0.03<br>ab	0.00<br>b	0.02<br>ab	0.14<br>a	0.03<br>ab	0.05<br>ab	0.03<br>ab	0.02<br>ab	0.13<br>ab	0.00<br>b	0.03<br>ab	0.09<br>ab	< 0.001
Energetic/<br>Excited	0.03<br>b	0.09<br>b	0.02<br>b	0.00<br>b	0.38<br>a	0.13<br>b	0.00<br>b	0.02<br>b	0.05<br>b	0.03<br>b	0.13<br>b	0.00<br>b	0.00<br>b	0.14<br>b	< 0.001
Enthusiastic/<br>Inspired	0.02<br>b	0.02<br>b	0.03<br>b	0.02<br>b	0.19<br>a	0.08<br>ab	0.09<br>ab	0.02<br>b	0.06<br>ab	0.02<br>b	0.11<br>ab	0.00<br>b	0.03<br>b	0.11<br>ab	< 0.001
Happy/<br>Satisfied	0.00<br>d	0.25<br>abc	0.17<br>bcd	0.00<br>d	0.33<br>ab	0.19<br>bcd	0.06<br>cd	0.03<br>d	0.09<br>cd	0.02<br>d	0.14<br>bcd	0.00<br>d	0.05<br>d	0.44<br>a	< 0.001
Secure/<br>At ease	0.00<br>c	0.20<br>ab	0.13<br>abc	0.02<br>c	0.02<br>c	0.09<br>abc	0.23<br>a	0.09<br>abc	0.03<br>c	0.00<br>c	0.03<br>c	0.00<br>c	0.03<br>c	0.06<br>bc	< 0.001
Relaxed/<br>Calm	0.03<br>c	0.17<br>bc	0.17<br>bc	0.00<br>c	0.02<br>c	0.16<br>bc	0.28<br>b	0.56<br>a	0.02<br>c	0.02<br>c	0.06<br>c	0.00<br>c	0.06<br>c	0.00<br>c	< 0.001
Passive/<br>Quiet	0.00<br>b	0.03<br>ab	0.14<br>a	0.00<br>b	0.00<br>b	0.05<br>ab	0.06<br>ab	0.14<br>a	0.02<br>b	0.00<br>b	0.03<br>ab	0.02<br>b	0.02<br>b	0.02<br>b	< 0.001
Dull/<br>Bored	0.00<br>c	0.08<br>bc	0.23<br>a	0.06<br>bc	0.05<br>bc	0.02<br>c	0.17<br>ab	0.06<br>bc	0.03<br>bc	0.06<br>bc	0.03<br>bc	0.00<br>c	0.11<br>abc	0.02<br>c	< 0.001
Blue/<br>Uninspired	0.11<br>a	0.02<br>a	0.03<br>a	0.08<br>a	0.00<br>a	0.05<br>a	0.05<br>a	0.00<br>a	0.05<br>a	0.06<br>a	0.09<br>a	0.03<br>a	0.09<br>a	0.03<br>a	0.067
Unhappy/<br>Dissatisfied	0.27<br>cde	0.02<br>f	0.03<br>f	0.69<br>a	0.00<br>f	0.05<br>ef	0.00<br>f	0.02<br>f	0.28<br>bcd	0.16<br>def	0.09<br>def	0.50<br>ab	0.42<br>bc	0.02<br>f	< 0.001
Tense/<br>Bothered	0.39<br>a	0.02<br>c	0.02<br>c	0.11<br>bc	0.00<br>c	0.02<br>c	0.00<br>c	0.02<br>c	0.23<br>ab	0.36<br>a	0.05<br>bc	0.38<br>a	0.11<br>bc	0.08<br>bc	< 0.001
Jittery/<br>Nervous	0.13<br>ab	0.05<br>b	0.00<br>b	0.03<br>b	0.02<br>b	0.05<br>b	0.02<br>b	0.00<br>b	0.11<br>b	0.27<br>a	0.11<br>b	0.08<br>b	0.05<br>b	0.00<br>b	< 0.001

[1]BA = baluts; BE = boiled eggs; BR = broccoli; BT = burnt toast; CA = cake; CO = coffee; CR = cooked rice; GT = green tea; HA = haggis; KI = Kimchi; MO = molded oranges; RA = rotten avocado; ST = steak

[2]The proportions with different letters within a row represent a significant difference at $p < 0.05$.

Supplementary Table S6. A contingency table of the proportions of citations by 105 participants across the 14 food image samples for individual pairs of emotion terms in the multiple response condition of Study 2

Emotion-related terms	BA	BE	BR	BT	CA	CO	CR	GT	GF	HA	KI	MO	SA	ST	*P*-value
Active/ Alert	0.14 cd	0.23 abcd	0.16 cd	0.08 d	0.47 a	0.44 ab	0.11 cd	0.09 d	0.20 bcd	0.14 cd	0.45 a	0.05 d	0.09 d	0.34 abc	< 0.001
Energetic/ Excited	0.05 de	0.22 cde	0.13 de	0.05 de	0.70 a	0.47 ab	0.16 de	0.08 de	0.20 cde	0.11 de	0.28 bcd	0.00 e	0.09 de	0.42 bc	< 0.001
Enthusiastic/ Inspired	0.03 d	0.17 bcd	0.22 bcd	0.02 d	0.58 a	0.34 bc	0.13 cd	0.11 d	0.14 cd	0.06 d	0.39 ab	0.02 d	0.06 d	0.34 bc	< 0.001
Happy/ Satisfied	0.02 ef	0.61 ab	0.45 abc	0.00 f	0.70 a	0.50 abc	0.44 abc	0.39 bcd	0.14 def	0.06 ef	0.28 cde	0.00 f	0.14 def	0.67 A	< 0.001
Secure/ At ease	0.02 e	0.40 ab	0.48 a	0.02 e	0.09 de	0.33 abcd	0.50 a	0.53 a	0.09 de	0.00 e	0.17 bcde	0.00 e	0.11 cde	0.34 abc	< 0.001
Relaxed/ Calm	0.00 d	0.50 abc	0.56 ab	0.03 d	0.14 d	0.42 bc	0.61 ab	0.72 a	0.05 d	0.03 d	0.11 d	0.02 d	0.11 d	0.25 cd	< 0.001
Passive/ Quiet	0.08 c	0.16 bc	0.22 bc	0.05 c	0.06 c	0.14 bc	0.28 ab	0.47 a	0.06 c	0.03 c	0.09 bc	0.03 c	0.11 ab	0.03 c	< 0.001
Dull/ Bored	0.05 b	0.11 ab	0.20 ab	0.16 ab	0.05 b	0.03 b	0.28 a	0.11 ab	0.03 b	0.13 ab	0.05 b	0.13 ab	0.14 ab	0.03 b	< 0.001
Blue/ Uninspired	0.16 bcd	0.08 cd	0.05 d	0.38 a	0.00 d	0.02 d	0.09 cd	0.06 cd	0.17 bcd	0.16 bcd	0.03 d	0.25 abc	0.31 ab	0.02 d	< 0.001
Unhappy/ Dissatisfied	0.64 bc	0.05 e	0.03 e	0.89 ab	0.06 e	0.06 e	0.03 e	0.05 e	0.41 cd	0.41 cd	0.17 de	0.92 a	0.56 c	0.03 e	< 0.001
Tense/ Bothered	0.59 a	0.03 c	0.00 c	0.45 a	0.00 c	0.02 c	0.00 c	0.03 c	0.39 ab	0.45 a	0.16 bc	0.58 a	0.41 a	0.08 c	< 0.001
Jittery/ Nervous	0.53 a	0.00 d	0.00 d	0.17 bcd	0.00 d	0.13 cd	0.00 d	0.02 d	0.39 ab	0.48 a	0.16 cd	0.31 abc	0.16 cd	0.06 d	< 0.001

[1]BA = baluts; BE = boiled eggs; BR = broccoli; BT = burnt toast; CA = cake; CO = coffee; CR = cooked rice; GT = green tea; HA = haggis; KI = Kimchi; MO = molded oranges; RA = rotten avocado; ST = steak

[2]The proportions with different letters within a row represent a significant difference at $p < 0.05$.

Supplementary Figure S1. Fourteen food image samples[1] used in this study

Name	Image	Name	Image
Balut	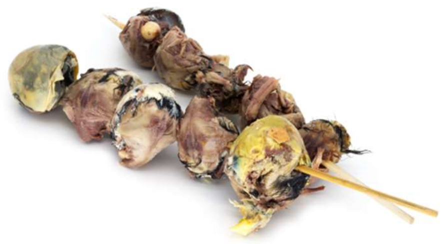	Unpeeled boiled egg and half eggs (boiled eggs)	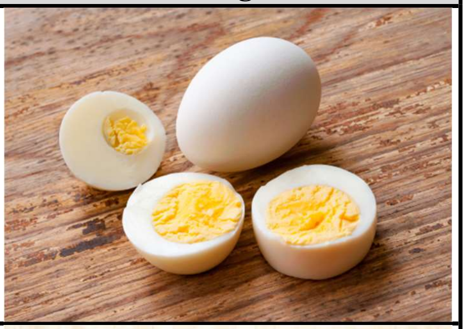
Birthday cake	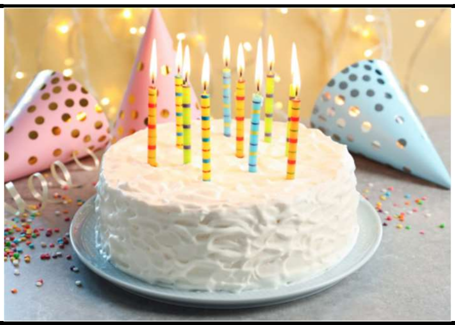	Grilled saury (grilled fishes)	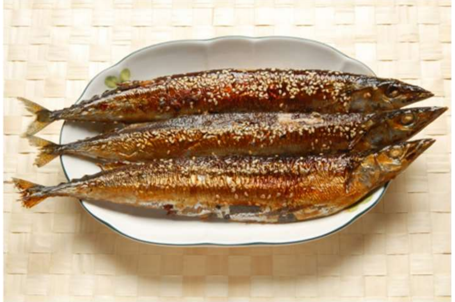
Brewed coffee (coffee)	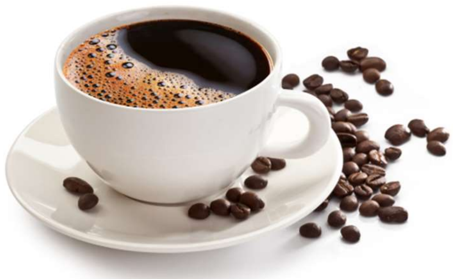	Scottish haggis (haggis)	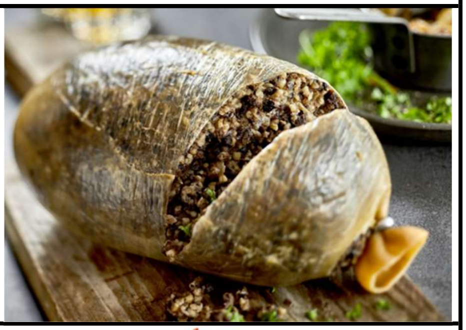
Brewed green tea (green tea)	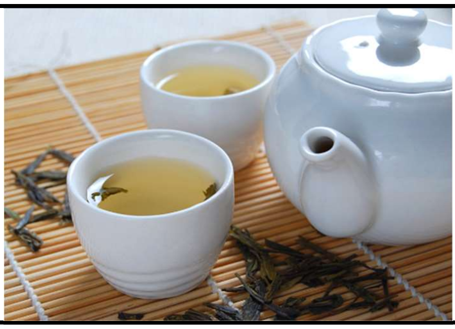	Kimchi	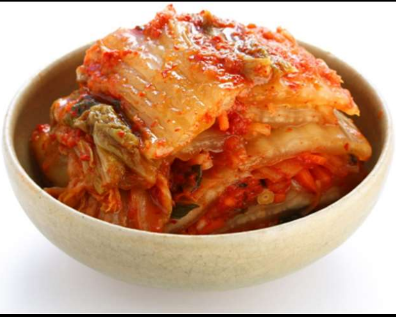
Steamed and sliced broccoli (broccoli)	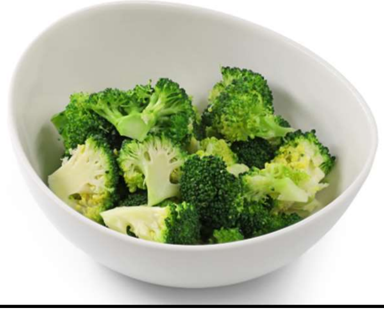	Molded oranges	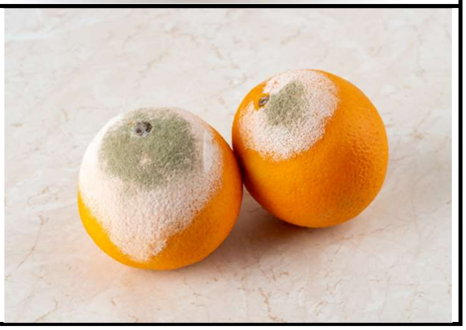

Supplementary Figure S1. Fourteen food image samples[1] used in this study (Continued)

Name	Image	Name	Image
Burnt toast bread slices (burnt toast)	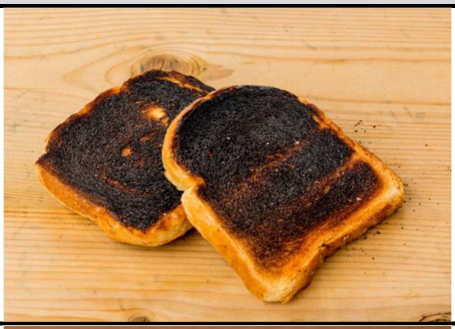	Half rotten avocado (rotten avocado)	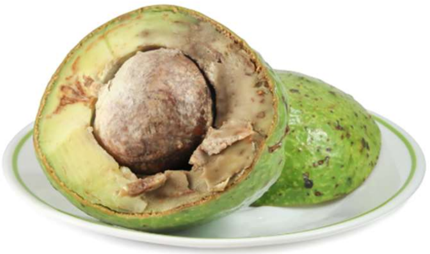
Cooked rice	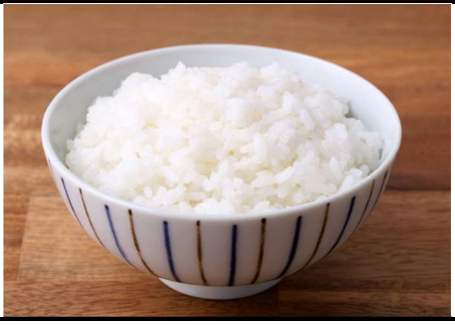	Beef steak (steak)	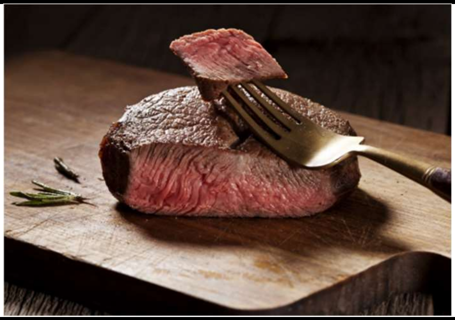

[1]All image samples were purchased and adapted with permission from a commercial web provider (Dreamstime, Brentwood, TN, USA).
